# Beneficial effect of LDL-apheresis in refractory nephrotic syndrome

**DOI:** 10.1007/s10157-013-0930-5

**Published:** 2014-02-18

**Authors:** Eri Muso

**Affiliations:** Center of nephrology and Urology, Division of Nephrology and Dialysis, Kitano Hospital, The Tazuke Kofukai Medical Research Institute, 2-4-20 Ohgimachi, Kita-ku, Osaka, 530-8480 Japan

**Keywords:** LDL-apheresis, Nephrotic syndrome, Secondary hyperlipidemia, Lipid nephrotoxicity, Cohort study

## Abstract

LDL-apheresis is a method to correct dyslipidemia rapidly. It is expected to alleviate the tissue toxicity of persistent dyslipidemia in not only primary, but also in secondary dyslipidemia associated with refractory nephrotic syndrome, and to have a protective effect against glomerular and tubular injury as expected in atherosclerosis. In addition, the effectiveness of LDL-apheresis to promote the remission of nephrotic syndrome has been recognized. In Japan, LDL-A to control hyperlipidemia in patients with refractory nephrotic syndrome associated with focal segmental glomerulosclerosis is covered by national health insurance. Here, the hypothetical mechanism behind its effect and the evidence for its effectiveness over a long period are reviewed.

## Introduction

Secondary dyslipidemia associated with refractory nephrotic syndrome (NS), typically that due to focal segmental glomerulosclerosis (FSGS), persists over a long period, causes the progression of cardiovascular disease with a course similar to arteriosclerotic lesions due to primary dyslipidemia, and exacerbates damage to affected glomeruli and renal tubules. LDL-apheresis (LDL-A) is a method to correct dyslipidemia rapidly. It is expected to alleviate the tissue toxicity of persistent dyslipidemia in this disease and to have a protective effect on the kidney. In addition, the effectiveness of apheresis therapy including plasmapheresis to promote the remission of NS has been recognized [1], but that of LDL-A has been suggested not necessarily to be due to the correction of abnormal lipid levels. At present, in Japan, LDL-A to control hyperlipidemia in patients with refractory NS associated with focal glomerulosclerosis FSGS is covered by national health insurance up to 12 times over 3 months, but clarification of the mechanism of the effect of this treatment and evidence for its effectiveness to maintain remission over a long period have been insufficient. Prospective cohort studies are being carried out, leading to the accumulation of evidence on its efficacy and clarification of cases in which the therapy is expected to be effective.

## Definition of refractory NS and characteristics of causative disorders

The international and Japanese diagnostic criteria for NS are nearly the same. Urinary excretion of protein >3.5 g/day, together with serum albumin at 3 g/day or less or serum total protein level of 6 g/day or less (these are essential diagnostic conditions), is expected to be maintained in association with edema and hypercholesterolemia (not essential items). Concerning the criteria of remission, in Japan, categories of type I and II incomplete remission (ICR) have been established, in addition to the international criteria of urinary excretion of protein at 1 g/day or less and 1–3.5 g/day, respectively. In Japan, refractory NS is defined as an inability to achieve type I ICR or complete remission (CR) despite the continuation of various treatments over 6 months or longer. The outcome was internationally reported to have been significantly poorer in those who were not included in these categories than in those who were, based on a survey of a large number of patients in Japan, and these categories are in wide clinical use and have been retained in diagnostic and therapeutic guidelines. Of the 3 major disorders considered to be causes of primary NS, FSGS and membranous nephropathy (MN) may develop into refractory NS. The pathological clarification of FSGS has advanced recently, and the nephrotoxicity of dyslipidemia associated with this disease has been reported. LDL-A was initiated against this disease in particular.

## Mechanism of occurrence of hyperlipidemia in NS and tissue toxicity of lipids

**Fig. 1:**
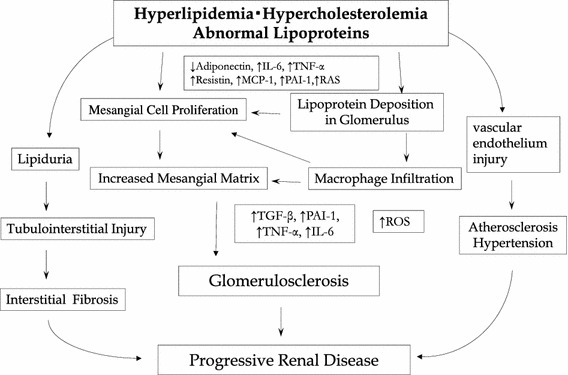
Lipid nephrotoxicity

Marked proteinuria due to NS causes severe hypoalbuminemia, promotes lipoprotein synthesis, and induces excessive albumin synthesis, resulting in hypercholesterolemia. Hypercholesterolemia is also promoted by metabolic disorders due to the loss of lipoprotein lipases that degrade LDL and VLDL cholesterols. LDL, particularly oxidized LDL, is incorporated by mesangial cells with scavenger receptors, forming foam cells. The foam cells and induced macrophages express various inflammatory cytokines and chemokines and cause tissue damage (Fig. 1) [2]. In addition, a large amount of protein leaks into the urine, but detached tubular cells that have absorbed fat are often observed. These reabsorbed excess lipids are considered to damage tissues by intensifying oxidative stress in the renal tubules [3]. Typical findings such as the frequent appearance of interstitial foam cells are observed in FSGS, in which dyslipidemia persists.

## Anti-nephropathic effect of the correction of hyperlipidemia associated with nephrotic syndrome

The secondary dyslipidemia mentioned above can be corrected by statins over a long period, but by LDL-A if an acute effect is expected. In LDL-A using a dextran sulfate column (Liposorber, Kaneka), which is prepared by coating porous Sepharose beads with dextran sulfate, LDL-cholesterol is adsorbed due to an electrostatic interaction between negatively charged dextran sulfate and positively charged apoprotein B on the surface of lipoprotein. VLDL and LDL are selectively adsorbed, but no HDL-cholesterol with ApoA or other plasma components including albumin is adsorbed. Liposorber can purify 3–4,000 ml of plasma in 2–3 h. When Sakai et al. first carried out this treatment for FSGS in 1988 in Japan, not only the correction of hyperlipidemia, but also rapid resolution of NS was observed, so coverage by national health insurance was extended to its application to FSGS with hyperlipidemia (LDL-cholesterol >250 mg/dl) in 1989.

## Evaluation of the mechanism of the effects of LDL-A (Table 1)

### Effects of adsorption of LDL, particularly oxidized LDL

The infiltration of lesions by macrophages induces cytokines and chemokines such as TNFα and IL-8, which are elevated in the serum of nephrotic patients, and causes inflammation and the activation of mesangial cells. LDL scavenger receptors present in these macrophages are likely to be hyperstimulated by an increase in LDL-cholesterol, particularly oxidized LDL, in the circulation. Evaluation of the effect of LDL-A on LPS-stimulated IL-8 production by peripheral monocytes by its comparison between before and after treatment revealed significant suppression of the responsiveness compared with that in healthy subjects before treatment, but this was significantly recovered after treatment [6]. This is considered to have been due to the recovery of macrophage function caused by the rapid elimination of LDL.

**Table 1:** Hypothetical mechanism of action of LDL-A on refractory NS

1. Direct effect of lipid (LDL, VLDL, oxLDL) adsorption
(1) Reduction of macrophage stimulation by ox-LDL
(2) Amelioration of macrophage dysfunction
(3) Reduction of inflammatory cytokine
2. Vasodilatory and anticoagulant effect by absorption of various pathogenic factors by dextran sulfate
(1) Reduction of fibrinogen and coagulatory factors
(2) Increase of VEGF/NO/bradykinin production, and decrease in thromboxane A2
(3) Absorption of vascular permeability factor
3. Enhancement of response immunosuppressant by amelioration of intracellular drug transport
(1) Amelioration of corticosteroid response
(2) Enhancement of transmembrane cyclosporine A transport via lipoprotein receptor
(3) Restore via inhibitory effects upon *MDR*-*1* gene expression

Inflammatory cytokines such as TNFα and IL-8 are significantly expressed in the blood of nephrotic patients, irrespective of the causative disease [4]. We observed that elevated IL-8 level was significantly reduced in the blood after comparison with that before several sessions of LDL-A (data not shown). This decrease in IL-8 derived from macrophages is considered to indicate the resolution of macrophage hyperstimulation.

### Adsorption of humoral factors responsible for NS

Savin et al. established an in vitro method to determine the albumin permeability of the glomeruli, and showed that the plasma of NS patients significantly increases the permeability. They also analyzed the patients’ plasma for humoral factors responsible for disease, and identified them as mildly acidic (pH 6.0) materials with a size of 30–50 kD [5]. However, the relationship between these factors and the occurrence of FSGS is unknown. In consideration of the involvement of these humoral factors, it is interesting that plasmapheresis and sometimes LDL-A, carried out in patients who showed recurrence after kidney transplantation, have been successful to a degree [6]. Another observation revealed that impaired IFN-γ production under IL12 stimulation of peripheral blood in persistent NS was restored by LDL-A, possibly through the removal of interfering serum factors [7].

### Recovery of cell sensitivity to drugs

In patients with CyA-sensitive FSGS, we have sometimes experienced that LDL-A recovered CyA effects at the same serum CyA concentration as had previously been refractory, especially in a relapse state. In terms of the mechanism behind this effect, CyA receptors taken into cells through binding with LDL are considered to have been saturated due to a high LDL level, preventing its absorption into the cells, but the rapid elimination of LDL is considered to have induced the recovery of the receptor function.

## Reports of evidence of therapeutic effects and trials

LDL-A was performed in 17 patients with FSGS not responding to steroid therapy that had been continued for 1 month or longer; the effect of the treatment and the remission rate were compared with those in 10 patients treated with steroids alone. Of the 17 patients who underwent LDL-A, CR was observed in 8 and type I ICR in 4; these results were significantly better than CR in 2 and type I ICR in 1 in the steroid alone group. More importantly, the time required to reduce proteinuria to 3.5 g/day, which is a criterion of NS, was markedly shorter in the LDL-A group. As a result, the steroid dose could be reduced earlier by the combination of steroid therapy and LDL-A. The remission rate was further increased in a follow-up study 2 years later, suggesting that the prognosis of even FSGS with refractory NS is favorable if remission can be achieved [8].

**Fig. 2:**
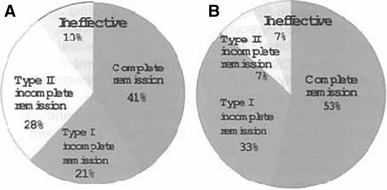
Retrospective survey of outcome of FGS patients with refractory NS treated by LDL-apheresis. Two-year outcome of 29 FSGS patients (**a**) and 5-year outcome of 15 patients (**b**) are shown

A survey concerning the long-term outcome was conducted primarily by the Japanese Society of Kidney and Lipids with the cooperation of 36 facilities, involving 94 patients with refractory nephrotic syndrome including 41 patients with FSGS and 28 patients with refractory minimal change nephrotic syndrome (MCNS) who underwent LDL-A in 1999 and thereafter [9]. The profiles of the FSGS and MCNS patients were as follows: male/female ratio: 24/16 and 14/14; mean age: 43 ± 19.6 and 35.7 ± 18.7 years; initial/recurrence ratio: 20/15 and 12/14; number of LDL-A trials: 8.25 ± 2.87 and 8.00 ± 5.57; and ratio between those who underwent kidney transplantation and those who did not: 7/24 and 0/25, respectively. In terms of the frequency of use of various drugs, steroids were used in 88 and 93 %, steroid pulse therapy was performed in 29 and 57 % (the prescription was the same as that before the initiation of LDL-A, except in 1 patient with MCNS), immunosuppressants were used in 41 and 46 %, CyA was employed in 29 and 36 %, and statins were used in 44 and 36 %, respectively. The percentages of patients who were included in the category of type I ICR after 2 years were 62 and 95 %, and those after 5 years were 87 and 80 %, respectively. Those of FSGS are shown in Figure 2. The response became more favorable as the time from the onset of NS to the introduction of LDL-A decreased.

Since the above studies were retrospective, a prospective cohort study (Prospective Observational Survey on the Long-Term Effects of LDL-A on Drug-Resistant Nephrotic Syndrome (POLARIS)) was initiated by the Japanese Society of Kidney and Lipids. In the preliminary analysis, almost the same remission rate was obtained, even in prospective study (under submission).

As shown in Table 2, on the basis of reported results of retrospective studies, LDL-A has been effective for inducing remission in nearly 50 % of patients with various diseases including FSGS that was refractory to NS, with a high level of safety. As noted in recently renewed guidelines for NS in Japan (2013), LDL-A should be selected as an option for the strategy to treat refractory NS.

**Table 2:** Clinical efficacy of LDL-apheresis for nephrotic syndrome (Summary of Clinical Studies before 2007)

	Muso et al. Nephron 2001 89 408–415	Stenvinkel et al. Eur J Clin Invest 2000 30 866–870	Yokoyama et al. Clin Nephrol 1998 50 1–7	Muso et al. NDT 1994 9 2257-264	Sakai et al. Jin To Touseki 1994 33 321–328	Hattori et al. Am J Kidney Dis 42 1121–1130
Study design	Study Group: prospective	Prospective	Retrospective	Retrospective	Prospective	Retrospective
No. of cases (control group)	Control group: retrospective 17 (10)	7 (none)	14 (none)	8 (none)	16 (none)	11 (none)
Primary disease (no. of cases)	FSGS (14/9) MCNS(3/1)	MN (3) MCNS(2) IgAGN (1)	FSGS (14) PSL resistant	FSGS(6) MCNS (1) MN + FSGS (1)	FSGS (13) MN (3)	FSGS (11) PSL, CyA resistant
No. of Treatment	2/w × 3 1/w × 6 Total 12	2/w × 3 1/w × 7 Total 13	2/w × 3 Total 6	2-13 7.3 (average)	2/w × 3 Total 6	2/w × 3 1/w × 6 Total 12
Concomitant treatment (no. of cases)	PSL 1.0 mg/kg	none (4) PSL(1) PSL + CyA (2)	PSL 0.8 mg/kg	PSL/pulse 1.0 mg/kg	PSL (14) immunosuppressant (10)	PSL 1.0 mg/kg
Clinical efficacy	Remission 9 Partial remission 4 no effect 4	Remission 2 Partial remission 4 no effect 1	Responded 8 no effect 6	Remission 4 Partial remission 1 no effect 3	Improved 7 Unchanged 3 Worsened 3 unjudgemental 3	Remission 5 Partial remission 2
Efficacy rate	76 %	86 %	57 %	63 %	FSGS 54 %	76 %
Summary	Reduced remission induction period	Increased serum albumin	Increased serum albumin Effective in younger age	Amelioration of ApoB deposition in glomerulus 5 in 6 cases	>50 % reduction of proteinuria in 9 cases	Effective in PSL resistant juvenile patients
